# Glyph guessing for ‘oo’ and ‘ee’: spatial frequency information in sound symbolic matching for ancient and unfamiliar scripts

**DOI:** 10.1098/rsos.170882

**Published:** 2017-09-13

**Authors:** Nora Turoman, Suzy J. Styles

**Affiliations:** 1 Department of Radiology, Centre Hospitalier Universitaire Vaudois (CHUV) and University of Lausanne (UniL), Rue du Bugnon 46, 1011 Lausanne, Switzerland; 2 Department of Clinical Neurosciences, Centre Hospitalier Universitaire Vaudois (CHUV) and University of Lausanne (UniL), Rue du Bugnon 46, 1011 Lausanne, Switzerland; 3 Division of Psychology, School of Humanities and Social Sciences, Nanyang Technological University, 14 Nanyang Drive, Singapore

**Keywords:** sound symbolism, cross-modal correspondences, writing systems, evolution of language, visual spatial frequency

## Abstract

In three experiments, we asked whether diverse scripts contain interpretable information about the speech sounds they represent. When presented with a pair of unfamiliar letters, adult readers correctly guess which is /i/ (the ‘ee’ sound in ‘feet’), and which is /u/ (the ‘oo’ sound in ‘shoe’) at rates higher than expected by chance, as shown in a large sample of Singaporean university students (Experiment 1) and replicated in a larger sample of international Internet users (Experiment 2). To uncover what properties of the letters contribute to different scripts' ‘guessability,’ we analysed the visual spatial frequencies in each letter (Experiment 3). We predicted that the lower spectral frequencies in the formants of the vowel /u/ would pattern with lower spatial frequencies in the corresponding letters. Instead, we found that across all spatial frequencies, the letter with more black/white cycles (i.e. more ink) was more likely to be guessed as /u/, and the larger the difference between the glyphs in a pair, the higher the script's guessability. We propose that diverse groups of humans across historical time and geographical space tend to employ similar iconic strategies for representing speech in visual form, and provide norms for letter pairs from 56 diverse scripts.

## 1. Introduction

What makes letters look the way they do? To some readers, it might seem intuitive that the sinuous shape of a letter like ‘s’ matches the dynamic continuity of the voiceless sibilant. To others, inconsistencies between spelling and pronunciation (e.g. ‘circus clowns’ /s, s, k, z/, respectively), and diversity among writing systems of the world might suggest that writing is arbitrary, and bear little systematic relation between written shapes and the sounds they represent.

There is growing recognition that human communication systems exhibit the property of iconicity, with symbolic representations expressing an aspect of meaning or a dimension of contrast in a way that is salient to the recipient [1,2]. For example, in the literature investigating linguistic sound symbolism, there is consensus that meaningful, systematic relationships can be found between the sounds of some words and their referents. Contrary to the principle of ‘arbitrariness’ [3], adults exhibit systematic preferences when matching particular novel word forms with imagined objects of different sizes (e.g. ‘mil’—small, ‘mal’—large, [4]) and line drawings of different shapes (‘takete’—jagged, ‘maluma’—rounded, [5]). Following pioneering demonstrations in the 1920s, linguistic sound symbolism has been widely reported [6–8], including in whole-of-lexicon investigations [9] and in a variety of implicit experimental and neuroimaging methods [10–15]. The ubiquity of the effect across ages and cultures [16–22] has led many to conclude that associations between linguistic sounds and visual features may be universal and/or innately predisposed [7,19,23].

Similarly, as has been pointed out previously [24], the sounds of canonically sound-symbolic phonemes tend to ‘go with’ the shape of their written letters. For example, Latin letters representing ‘soft’ phonemes (/b/ /m/ and /u/) tend to be wider, and more curved than Latin letters representing ‘sharp’ phonemes (/k/, /t/ and /i/) tend to be more angular. This alliance between letter shape and sound has led some to suggest that effects of linguistic sound symbolism previously observed in European studies may be driven by the shapes of written word forms [14,16], or by some combination of letter shape and phoneme sound [25–27]. Since ‘activation’ of letter representations is more or less automatic in literate adults, the relationship between visual form and phonological form is difficult to disentangle in these populations.

One response to this letter/sound entanglement has been work with populations with limited exposure to Latinate letters, including preliterate children [17–19], preverbal infants [21,22] and remote communities with limited prior exposure to Western cultural artefacts or writing [16]. In all but one reported study [28], the effect occurs regardless of language background and regardless of script exposure. By demonstrating that the *bouba–kiki* or *maluma–takete* effect replicates in non-Latinate populations, these studies effectively show that letter–shape associations are not necessary for cross-modal congruence between word forms and visual shapes.

However, none of the existing studies have experimentally addressed the question of why the relationship between letter shapes and sounds in Latin exists in the first place. Indeed, given that Latin letters are not required for a bouba/kiki effect, the direction of causation could go either way: The sound of /k/ might seem sharp *because of* the shape of the letter ‘k’ (shape-to-sound iconicity). Alternatively, the letter may be sharp *because it represents a sharp sound*—that is, script developers might have tried to create writing systems that captured salient sensory properties of the sounds they represented, by designing glyphs that would ‘go with’ their sounds (sound-to-shape iconicity). The ‘entanglement’ of the visual forms of letters with the sounds they represent has been noted previously [29,30]. Koriat & Levy [29] asked participants to rate vowel glyphs on the sensory dimensions of magnitude, brightness, hardness and length. The glyphs were from unrelated script families (Japanese Katakana and Devanagari) that were unfamiliar to the participants (Hebrew speakers). In their study, the glyphs were ranked across each visual dimension in the same order as had previously been documented for participants familiar with the Latin alphabet—the sequence /i, e, a, u, o/ represented increasing size, and decreasing brightness and hardness in all three scripts. This finding suggested a common cross-modal core for the visual representations of speech sounds in three scripts. To date, no studies have investigated whether a broader sample of writing systems of the world encodes this kind of sensory information systematically—in a way that might allow naive participants to guess letter identity—nor what visual properties might drive the effect.

The aim of the current series of experiments is to investigate whether humans across geographical space, and historical time create writing systems that encode salient properties of the speech sounds they represent, and to investigate physical properties of the writing systems which might give rise to these effects. If sound–shape correspondences underlying letter forms have their basis in shared systems of multisensory processing, then the visual encoding of speech sounds should apply to writing systems developed at earlier points in time and at geographically distant locations. We therefore designed an original stimulus set, consisting of letter pairs representing the high front unrounded vowel /i/ (the ‘ee’ vowel in ‘feet’) and the high back rounded vowel /u/ (the ‘oo’ vowel in ‘shoe’) in a variety of ancient and unfamiliar writing systems spanning recorded human history (see Material and methods). The /i/–/u/ pair provided a comparison between highly contrastive [31], high prevalence [32], isolated speech segments, uncomplicated by interactions between vowels and consonants (cf. [22,33]). The vowels /i/ and /u/ differ in backness (/i/: front; /u/: back), and in lip rounding (/i/ unround/retracted; /u/: rounded/protruded), and have been shown to differ in their sound-symbolic congruence patterns for curvy/spiky shapes (cf. [34–36]). Theorists have proposed different mechanisms for the mapping patterns of vowels: Ramachandran & Hubbard [7] proposed that the mirror system is responsible for mapping the visual features of lip rounding to curved forms; Ohala [37] has proposed that lip protrusion in primates generates acoustics consistent with a larger body size, as lengthening the vocal tract lowers the respective peak frequencies in vowel-sound spectra, i.e. the second and third formant. Regardless of mechanism, all accounts agree that /i/ tends to go with small/spiky shapes, and /u/ with large/curvy shapes. Here, we look at systems of human representation (writing systems), for their ability to capture the salient properties of the /i/–/u/ contrast.

**Figure 1.**
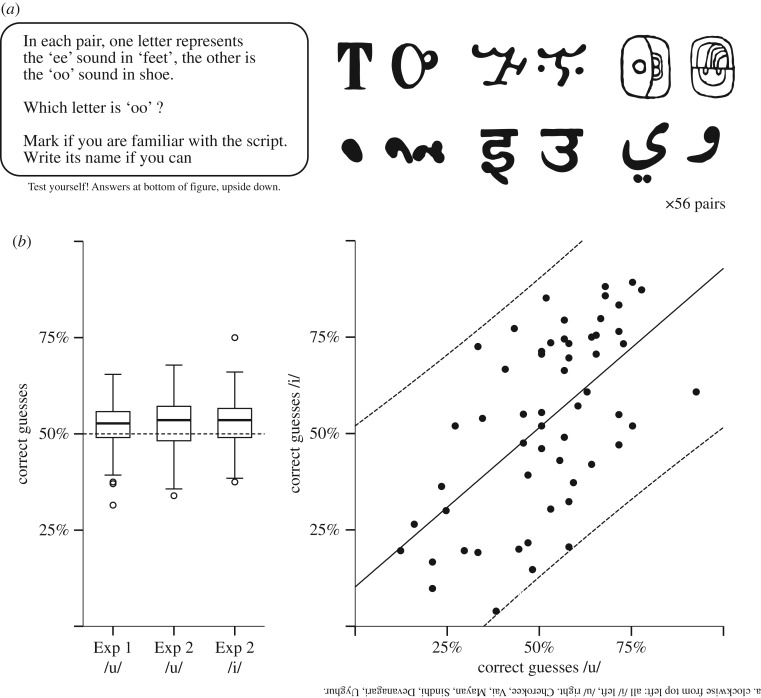
Glyph guessing. (*a*) Instructions and example letter pairs. (*b*) Box plots showing the distribution of correct guesses in two experiments where people guessed which letter in a pair was /u/ (Experiment 1, *N* = 98; Experiment 2, *N* = 81) and /i/ (Experiment 2, *N* = 102). Dashed line shows chance (50%). (*c*) Scatter plot showing the relationship between the mean rate of /i/ and /u/ guessing for Experiment 2, with each script shown (*N* = 56). Solid line shows correlation; dashed line shows 95% confidence level of mean.

To test whether participants could guess the identity of ancient and unfamiliar glyphs for /i/ and /u/, we developed a test containing 56 letter pairs. As illustrated in figure 1*a*, participants were informed that the letters represented the ‘ee’ sound in ‘feet’ and the ‘oo’ sound in ‘shoe’, and were asked to guess which letter in each pair was the ‘oo’. (The test sheet is available in the Open Science Framework (OSF) repository for Turoman & Styles [38]: https://osf.io/xufmd.) In a small-scale pilot study reported elsewhere [39], psychology students in a seminar class on language (*N* = 20) guessed with above chance accuracy, providing proof of concept for the larger, more formal studies reported here.

## 2. Experiment 1

Ninety-eight university students in Singapore filled in the paper form used in the pilot study. They were asked to guess which letter in each pair they thought was ‘oo’, and to mark any scripts they thought were familiar, and name them if they could. Since the goal of the study was to investigate responses to unfamiliar writing systems, we excluded any scripts that were marked as familiar and correctly named at the level of the individual. Following these exclusions (1.5% of all answers), participants guessed the correct letter for /u/ at levels higher than chance (*M* = 52.7%, s.d. = 6.16%, *t*_97_ = 84, *p *< 0.0001, *d* = 0.44, 1 − *β* = 0.996; figure 1*b* (left)). This study replicates results of the pilot study in a large, well-powered sample.

## 3. Experiment 2

To replicate and extend the results of Experiment 1 in a more diverse sample, Experiment 2 was implemented online via the Qualtrics survey platform. The online cohort was run in two batches, with the first group guessing which letter was /u/ and the second group guessing which letter was /i/. Following exclusion of familiar, correctly named scripts for each individual (1.1%) people in the online cohort guessed /i/ and /u/ phonemes at rates better than would be predicted by chance (/u/: *M* = 52.3%, s.d. = 7.3%, *t*_80_ = 2.78, *p* = 0.007, *d* = 0.31, 1 − *β* = 0.87; /i/: *M* = 53.2%, s.d. = 6.4%, *t*_101_ = 5.1, *p *< 0.001, *d* = 0.50, 1 − *β* = 0.9996) with no observable difference between the two participant groups (*t*_181_ = 0.94, *p* = 0.35, *d* = 0.14; figure 1*b* (left)). For each script, the proportion of correct /i/ guesses and /u/ guesses was moderately correlated (*r* = 0.60, *p *< 0.0001), meaning that if one item in a pair was typically guessed to be /i/ and the other was typically guessed to be /u/ (and vice versa). However, the spread of results is quite broad, suggesting some variability across items and individuals (figure 1*b* (right)).

This study replicates the findings of Experiment 1 in a larger, more culturally diverse sample, providing further evidence that people are better than chance at guessing the identity of highly contrastive phonological pairs in ancient and unfamiliar scripts, and that both the /i/ and the /u/ vowels contribute to the observed effect. These results suggests that modern Internet users share some kind of sensory mapping between speech sounds and visual shapes with temporally and geographically distant humans, such that some characteristics of the sounds can be extracted from the letter shapes, at rates better than would be predicted by chance.

The results of Experiment 2 are also presented as norms for the letter pairs in the Open Science Repository for this project (https://osf.io/xufmd).

## 4. What drives sound-symbolic guessing?

In both Experiment 1 and Experiment 2, some scripts had a higher rate of correct letter guesses than others, with participants across experiments showing high agreement (correlation between script-scores for /u/ in Experiment 1 and Experiment 2: *r* = 0.89, *p *< 0.001). In an informal visual inspection of the pilot study, we had previously observed that scripts with the highest rates of correct guesses were those where /u/ tended to exhibit curvature, width, complexity and low-positioned detail, and /i/ tended to exhibit straightness, tallness, simplicity and high-positioned detail [39]. Conversely, scripts with the lowest rates of correct guesses were those that exhibited the opposite pattern. These patterns are in line with previously reported /i/ and /u/ correspondences [20,33,35,36], and led us to believe that measurable visual properties of the letters could be responsible for the observed effects.

In the cross-modal matching work of Evans & Triesman [40], high-pitched sounds generate reaction time facilitation for small visual objects—an effect which has also been observed in young children [41]. Since a small visual object activates a small area in the receptive field of the retina, it therefore represents high-spatial frequency information. Evans & Triesman further demonstrated that pitch had the same facilitatory effect on high-spatial frequency stimuli as on small-sized stimuli, confirming a low-level cross-modal link between visual spatial frequency and acoustic fundamental frequency (pitch). Ohala's [37] proposal that small body size is signalled by the higher *spectral* frequencies of formants in lip-retracted vowels (e.g. /i/) when compared with lip-protruded vowels (e.g. /u/) suggests that size may be similarly mapped not only to fundamental frequency information (pitch), but also to spectral frequency information (vowel formants). Hence, for the visual analysis that follows, we investigated whether the selection of which glyph was /i/ and which was /u/ could be driven by the spatial frequency information in the letters. We therefore investigated if scripts that maximized spatial frequency distribution differences were the ones that attracted the highest proportion of correct guesses.

## 5. Experiment 3

In this exploratory analysis, we extracted the spatial frequency distribution of each letter stimulus and compared the guessability of each script with the spatial frequency information. We considered two spatial frequency dimensions: peak spatial frequency (differences in the *x*-axis) and detection rate (differences in the *y*-axis). Following the logic of EEG difference waves, we computed the difference in detection rate at each measured spatial frequency (/u/ minus /i/), to give a measure of how much the SF differed between letters in each script.

**Figure 2.**
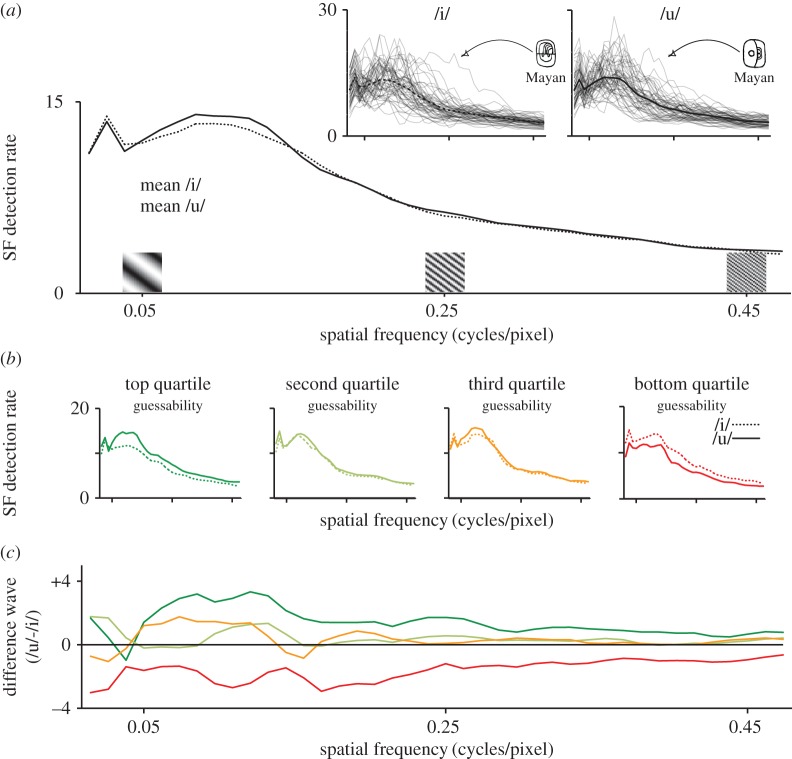
Spatial frequency distribution for glyphs. (*a*) Mean detection rate for the number of black/white cycles detected at different visual spatial frequencies, as measured from each glyph, with raw data for each glyph shown in inset. Spatial frequencies are illustrated, and one outlier script (Mayan) is highlighted. (*b*) Mean spatial frequencies for scripts in the four quartiles of guessability, as established in Experiment 2 (mean of /i/ and /u/ guessing). (*c*) Difference waves showing the difference in the detection rate between glyphs in the same script (/u/ minus /i/) separated by the scripts' guessability quartile.

Figure 2*a* shows the distributions of the mean spatial frequency distribution for /i/ and /u/ glyphs, with the raw data from each letter shown in the inset. The distributions show similar profiles across the spatial frequency range, as would be expected from stimuli where each letter in a pair has similar line thickness. In this figure, it is clear that the peak spatial frequency for /i/ glyphs and for /u/ glyphs is similar, with no meaningful difference between /i/ and /u/ modal spatial frequencies (/i/—Peak SF: *M* = 0.087, s.d. = 0.047; /u/—Peak SF: *M* = 0.086; s.d. = 0.040, *t*_55_ = 0.32, *p* = 0.75, correlation between glyph-pairs: *r* = 0.69, *p *< 0.0001).

Figure 2*b* shows the observed values for /i/ and /u/ pairs, for scripts at different levels of guessability, and figure 2*c* presents the grand average difference waves for each quartile. It is clear that detection rate differences between /i/ and /u/ are different for scripts at different levels of guessability: the medians of the difference waves are significantly different for scripts in each quartile (*F*_3,55_ = 19.40, *p* < 0.0001), with the more-guessable scripts in the top three quartiles exhibiting higher detection rates for the /u/ glyph than for /i/ glyph within a script pair, and the lowest quartile showing an inverted pattern, with higher detection rates for /i/ than for /u/ (top quartile: *M* = 1.10, s.d. = 0.91; second quartile: *M* = 0.36 s.d. = 0.70; third quartile: *M* = 0.27; s.d. = 0.85; bottom quartile: *M* = −1.26, s.d. = 0.88; Tukey's HSD: all comparisons with bottom quartile *p *< 0.0001; second versus third: *p* = 0.99; top versus second: *p* = 0.11). This finding shows that for the most-guessable scripts, more black/white transitions were detected across the spatial frequency range for /u/ than for /i/, indicating more marks/longer lines with similar graphical properties (i.e. similar line thickness).

**Figure 3.**
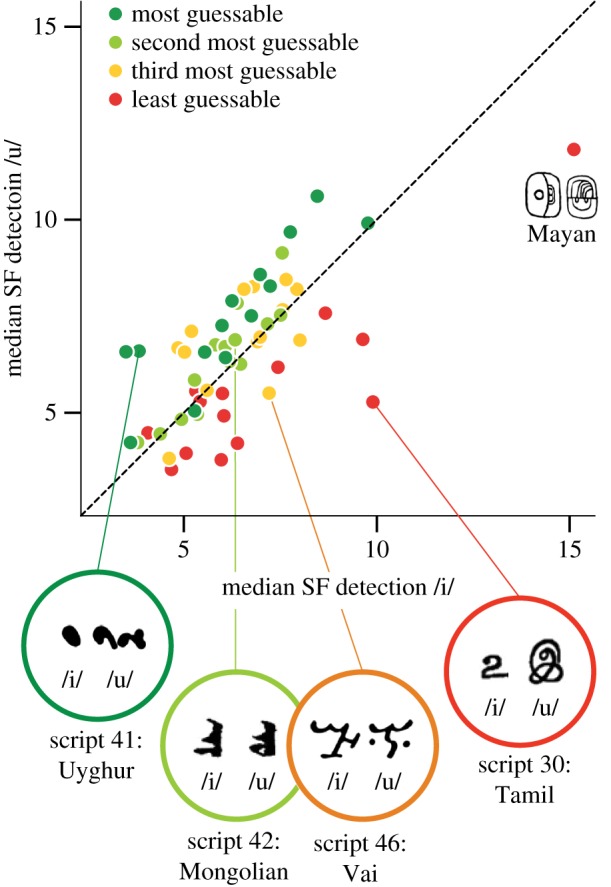
Scatterplot of median detection rates for /i/ and /u/ glyphs, shown for each glyph, with the script's guessability quartiles shown in colour. Dashed line indicates equivalence. Above the line, scripts have higher SF detection for /u/ than for /i/. Data from all 56 scripts shown, with outlier (Mayan) highlighted. Examples of scripts with a large positive difference (Uyghur), a small difference (Mongolian, Vai) and a large negative difference (Tamil) are shown.

Figure 3 shows the tight relationship between the SF detection rate for glyphs within a script pair (*r*_56_ = 0.69, *p* < 0.0001, *r*^2^ = 0.47). In this figure, we give an example of a script with a higher SF detection rate for /u/ than for /i/ (Uyghur), two scripts with similar detection rates for /u/ and /i/ (Vai, Mongolian) and a script with higher detection rates for /i/ than for /u/ (Tamil). In this figure, an additional outlier (Mayan) exhibited a notably high median SF detection rate for both /i/ and /u/, which is evidently a combination of thin lines and highly detailed characters.

**Figure 4.**
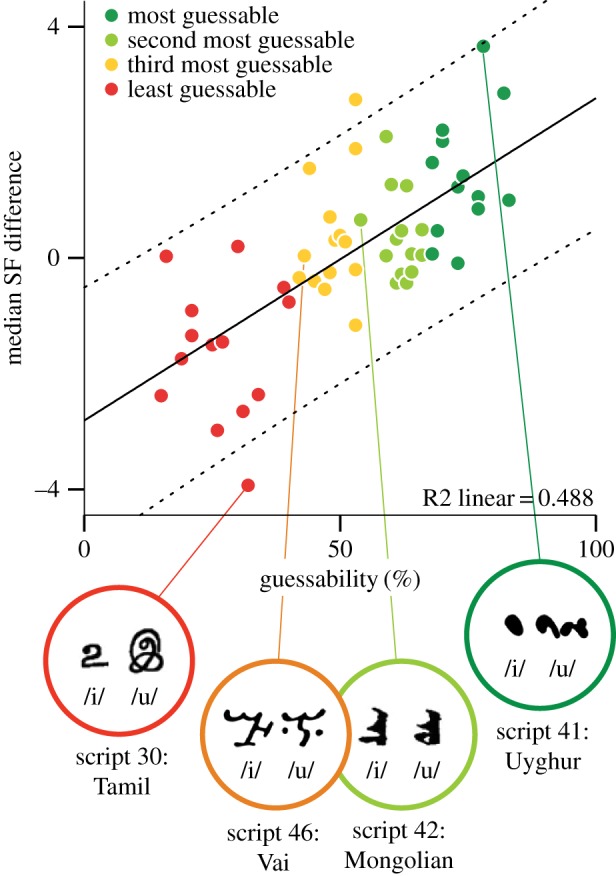
Scatterplot of each script's guessability and median of the SF difference wave (the difference in detection of black/white cycles at difference spatial frequencies: /u/ minus /i/). Solid line shows linear relationship. Dashed lines show 95% confidence interval of mean. Data from all 56 scripts shown. Examples of scripts with a large positive difference (Uyghur), a small difference (Vai, Mongolian) and a large negative difference (Tamil) are shown.

Figure 4 shows a linear relationship between guessability and detection rate difference between letters (*r*_56_ = 0.69, *p *< 0.0001, *r*^2^ = 0.49): the larger the difference (/u/ greater than /i/) the more likely the script was to be guessed correctly—with large differences resulting in high guessability scores (e.g. Uyghur), inverse differences resulting in incorrect guesses (e.g. Tamil) and small differences resulting in chance responding (e.g. Vai), as illustrated.

These exploratory analyses reveal that guessability for an /i/–/u/ letter pair does not rely on the peak spatial frequency of the letters as we initially predicted, but is sensitive to the overall number of black/white transitions (the amount of ink) detected within a glyph. When /u/ includes greater line length and/or complexity than /i/, the letter pair becomes more guessable. This finding makes sense, given that writing systems tend to develop stylistic conformity within their glyph sets, resulting in a similar graphical structure for different letters within a script. Firstly, in terms of mechanical conformity, glyphs within a single writing system are created using the same tools and materials (e.g. Cuneiform wedged impressions in clay, Egyptian reed pens on papyrus and Japanese brush strokes on paper), resulting in similar mark thickness within a given medium. Secondly, for coherence and legibility within the single text, glyphs typically fall into a fixed area between two lines, resulting in roughly similar glyph size. Within these constraints, it is clearly possible to create visual diversity among glyphs. Here, we observe one dimension that can convey iconic meaning is line length/complexity for glyphs representing speech sounds contrasting in the height of their second and third formants. Although there may be multiple strategies at play in the visual representation of speech sounds, this study therefore provides the first suggestion of a possible iconic link between spectral frequency information in speech and spatial frequency information in visual representations of speech.

## 6. Discussion

In a series of experimental investigations, we observed that modern participants were able to guess which of two ancient or unfamiliar letters represented which of two contrasting speech sounds (/i/ and /u/), at rates which were higher than would be predicted by chance. Although the deviation from chance is small (52–53%), we replicated the effect in three different groups of participants, each of which represented a high-powered sample. As this result was obtained after excluding scripts that participants recognized, these guesses must be driven by something other than knowledge of the letter shapes and sounds. We propose that basic properties of the sensory system are involved in cross-modal matching between speech sounds and shapes. Furthermore, these results suggest that humans across geographical space and historical time have shared audio–visual correspondences, which may have guided the creation of written letter forms. These glyphs encode salient sound contrasts in a way that tends to be recognizable even for people unfamiliar with the writing system in question. Although these historical experiments have not always been successful, on the whole, humans tend to invent writing systems that humans can interpret. We propose that one of the interpretation strategies invoked is visual complexity, with a general pattern that when glyphs for /u/ contain more lines than /i/, the letter mappings are more guessable.

### 6.1. Origins of letter–sound matching

Linguistic audio–visual correspondences are widely considered to be part of a general pattern of cross-modal correspondences between auditory and visual stimulus features, for example, pitch and visual elevation, spatial frequency and size [40,42], pitch and brightness, lightness and shape (angularity/roundedness), and loudness and brightness [43]. Such correspondences have been observed very early in life (e.g. pitch–lightness in 30–36 month olds: [41], loudness and brightness in 20- to 30-day-old infants: [44], pitch and visual elevation in three to four month olds: [45], pitch and size in four month olds: [46], for review, see [47]) and even in non-human populations (pitch and lightness in chimpanzees as in humans: [48]). This reinforces the view that audio–visual cross-modal correspondences are rooted in the physiology of our sensory systems. Indeed, emerging evidence suggests that the neurobiological substrates of the bouba/kiki effect appear to be linked to brain regions associated with multisensory processing [49], and neural processes involved in object processing [10].

Many researchers agree that cross-modal integration arises out of multisensory neurons tuning to the statistical regularities of the environment [50]. For example, larger resonating bodies typically carry lower-pitched sounds, while smaller bodies carry higher-pitched sounds. This correspondence occurs consistently for animals and objects [51–55], giving substantial environmental support for learning sensory congruence (i.e. low pitch = large) and generalizing it to new contexts. The case for natural languages is somewhat more complicated, as it is difficult to untangle whether sensory mappings between linguistic sounds and their referents arise out of experience with what particular words mean, or as an offshoot of general multisensory processing established by environmental exposure. In our study, the correspondence between spatial frequency cycles (line length/complexity) and lower frequency components in an auditory signal (lower F2 and F3 for /u/ when compared with /i/) is consistent with environmental regularities such as large body size/low pitch, as proposed by Ohala [37]. Furthermore, in the work of Evans & Triesman [40], high-pitched sounds generate reaction time facilitation for visual objects with high-spatial frequency, further confirming a low-level cross-modal link between visual spatial frequency and acoustic frequency (in this case pitch). We therefore propose that this effect is an extension of the congruence patterns exhibited by the general sensory processing system—an effect that may not need to be acquired through exposure to written language.

It should be noted that the simple measurement of spatial frequency cycles may be picking up low-level sensory information from a variety of iconic strategies (e.g. small versus large; simple versus complex), and this visual feature may be just one of many that are contrasted in glyphs in different writing systems. For example, some glyph-pairs may exhibit more contrast for curved versus angular forms, high versus low positioning or vertical versus horizontal elements, without any attendant difference in observed visual spatial frequency. Indeed, the wide spread of responses across this glyph set, and the spread of the data in the critical comparison (figure 4) suggests that multiple factors may be at play in judgements about individual glyph-pairs. However, having uncovered a novel link between visual spatial frequency and representation of speech sounds provides a new dimension for future investigations into iconicity in human communication systems. The straightforward measurement properties of spatial frequency can also be used to create new generations of stimuli for future investigations into cross-modal linkages both within and outside the domain of language.

This study investigated only the single vowels /i/ and /u/, meaning that more or less systematicity may be evident in other sounds/letters in world writing systems. For example, it may well be the case that sound-symbolic guessing for consonant glyphs may be stronger than it is for vowel glyphs, a finding that would be consistent with experimental pseudoword shape-matching tasks [33,36]. As it stands, the choice of two highly contrastive ‘bare’ vowels allows the smallest unit of comparison that is utterable, implemented using high prevalence, highly contrastive phonemes that are canonical for these kinds of effects [56]. Despite the narrow phonological range, this study provides proof of concept for further (e.g. whole of writing system) investigations. For example, it may well be the case that some writing systems are more iconic than others (at the level of the whole writing system), rather than at the level of individual pairs. On the other hand, it may be that some sound pairs are uniquely iconic due to their dimensional contrastiveness, and that most languages do more or less well on pairs of this kind. These details have yet to be established by future work.

One further caveat on the interpretation of the findings is that the response pattern we observe may not reflect an iconic strategy in glyph generation, but may instead be driven by the task demands of making massed decisions about a large number of visual stimuli in a single testing setting, for participants with a particular language/script background (i.e. all participants took the test in English and are therefore familiar with the visual properties of the letters ‘i’ and ‘u’). It has been pointed out previously that massed testing of this kind could induce task-specific decision strategies that may be driven by the structure of the items in the test set. For example, if different letters pairs used different iconic strategies, these effects may be attenuated by conflicting response strategies [30]. In our case, letters differ substantially across the full set on a number of visual dimensions (including peak visual spatial frequency, size, curvature, angularity, line width and orientation). Within a letter pair, these differences are more or less controlled by the stylistics of the script, leaving differences in observed spatial frequency (ink density), for most scripts. This could mean that the use of multiple scripts lead participants to pay more attention to the ink density characteristics than to other salient differences between letter pairs. Alternatively, they could have settled on this response strategy by analogy to Latin letters. Future iterations could use smaller subsets of the glyph-pairs (one-shot tests), to wash out conflicting response strategies, or test whether the visual properties identified here have a more general role in multisensory processing, to clarify the source of the observed effects.

Previous research has begun to document cross-cultural differences in linguistic sound symbolism [28,56], as well as in audio–visual cross-modal mappings related to linguistic metaphor [46,57,58]. For instance, in at least two cases, the well-known matches between pseudoword pairs like bouba/kiki and maluma/takete do not show the expected pattern of audio–visual correspondence in languages where the test items do not match the sound structure of the language (see [56] for extended discussion). Also, in cross-modal matching between pitch and the visual dimensions of height or thickness (German: ‘high’ versus ‘low’ pitch; Farsi and Turkish: ‘thin’ versus ‘thick’ pitch), prelinguistic infants show sensitivity to mapping strategies that are used by a variety of cultures [46], while adults tend to prefer to match according to the dominant linguistic metaphor in their culture [57]. These adaptations of the linkages between auditory and visual processing represent a kind of multisensory perceptual narrowing analogous to the ‘tuning’ processes well known to occur in the speech perception of young infants [59–61]. This experience-dependent developmental trajectory from general to culture-specific audio–visual matching may help to account for differences between universal patterns of sound symbolism and conventionalized, culturally bound ones. A similar chain of reasoning exists in the feedback theory of Taylor & Taylor [62,63], whereby shared subjective experiences of sound-symbolic matching (subjective sound symbolism) may become conventionalized in a speech community over time (objective sound symbolism).

In the context of the present study, although our participants report culturally and linguistically diverse backgrounds (especially in Experiment 2), all were familiar with the conventions of the English language, and thus shared some elements of cultural background. The observed pattern of responses could represent more or less WEIRD responding (i.e. responses from Western, Educated, Industrialized, Rich and Democratic nations [64]) due to elements of shared culture (although note that participants in Experiment 1 were from southeast Asia). Importantly, Latin letters canonically used to represent the target vowels in English also show an observed spatial frequency difference in the same direction as was found for highly guessable scripts in this study. It is therefore not possible to disentangle whether the spatial frequency metric was a driver of sound-symbolic matching due to a low-level sensory bias, or due to the participants' experience with the English language visual contrast. Such confounds between general patterns of sensory matching and culturally mediated patterns of sound symbolism have been noted elsewhere (e.g. [16,19,21,45,65,66]). One way to concretely untangle these possibilities in the future will be to investigate whether participants without English script knowledge show the same matching biases (e.g. illiterate populations/literate participants with no knowledge of the Latin script/infants).

Despite these caveats, our data show that participants tend to extract discriminative information from the majority of letter pairs, resulting in above chance guessing, but the rates of guessability for different letter pairs were highly variable. In exploratory analyses, guessability was linked to difference in the frequency of observations of spatial frequency cycles, which suggests that line length/complexity is a new dimension for cross-modal mapping.

### 6.2. Letters as multisensory objects

From an evolutionary perspective, writing is a relatively recent technology, meaning that the links between text and speech must be subserved by neural functions originally adapted for other purposes [67]. This mismatch between novel functions and evolved systems may make letter representations acutely fragile to disruption from subtle differences in multisensory processing [7], with grapheme-induced synaesthesia representing disregulated and/or hyperactive multisensory processing [68–71], and letter decoding difficulties well known in dyslexia representing disregulated and/or hypoactive multisensory processing [72–78]. In this study, we provide a measurable source of cross-modal activity linking acoustic properties of speech sounds and visual properties of glyphs. Research into individual differences may reveal further modulation of these effects in different subsets of readers.

### 6.3. Iconicity and arbitrariness

So why does our task ‘work’, but not well in all cases? Iconicity is known to enhance both learnability [17,18] and the efficiency of recognizing previously learned symbols [79], even under conditions of visual masking [13]. Given these properties, a few logical consequences follow: first, if more-iconic writing systems are more learnable, learnability may influence script uptake, meaning that scripts with higher rates of iconicity may translate into higher rates of literacy. This argument is analogous to arguments about writing system transparency—if a system is easier to acquire in the early stages of script encounter, this may follow through to higher rates of effective literacy in the general population; second, if writing is produced by hand, and teaching and learning are ‘unsupervised’ by linguistic authorities, glyphs may exhibit a selective pressure to become more iconic over time, especially if the cycle from learner to teacher is short. The argument here is analogous to the acquisition cycle in contact languages, where rates of change in pidgin/creole languages are rapid when children learn from each other, and there is no authority on ‘correct’ usage to slow down the rate of change. However, despite both of these principles being logical outcomes of iterated learning and transmission, neither will occur spontaneously if there are structural barriers to script accessibility, transmission or change (e.g. literacy only available to wealthy; lack of peer-to-peer teaching and/or taboos against non-standard script usage). We believe that one of the reasons our effect is not more prevalent is that scripts are not universally adapted to their own transmission. Rather, they rely on complex patterns of cultural transmission in formalized settings (i.e. schooling).

Finally, it is relevant to note that if all acoustic contrasts were encoded using a single dimension of sensory iconicity, items would become easily confusable. A similar point has been made previously by Dingemanse *et al.* [2], who propose that iconicity serves its primary purpose in grouping together similar items (e.g. category learning), while arbitrariness allows further fine-grained differentiation between similar items (i.e. item learning), an effect which has been experimentally demonstrated by Ković *et al.* [80]. Hence, if a single iconic feature (e.g. line length) was used to encode the difference between front vowels (/i/,/e/) and back vowels (/o/, /u/), it might be easy to tell the letter /i/ (less ink) from /o/ (more ink), but difficult to discriminate /i/ (less ink) from /e/ (also less ink). For this reason, writing systems probably use more than one visual strategy to differentiate their letters. However, if the system contains some degree of arbitrariness, then discrimination between similar items becomes less difficult. In our study, while we observed that different ink-volume was a common strategy to differentiate the /i/ versus /u/ vowel contrast, variance such as that observed between different letter pairs in the test set (e.g. figure 4: Uyghur, Sindhi) suggests a large degree of arbitrariness in this visual feature.

### 6.4. Further directions

For communities with unwritten languages, unpacking the visual properties of speech–sound association in individual languages could help those communities develop writing systems that are uniquely ‘tuned’ to the sensory properties of the language, potentially making them easier to acquire and disseminate in small communities. In script development work, we therefore suggest that line length/complexity be considered as a useful visual property for helping to discriminate speech sounds with high- versus low-frequency spectral components (e.g. /i/ from /u/).

This study provides norms for letter-pair guessability, as well as measured spatial frequency information for the individual letters in a variety of writing systems that are likely to be unfamiliar to the majority of contemporary readers in the West. The full stimulus collection and dataset can be found in the OSF repository [38] for this project (https://osf.io/xufmd). These collections are therefore a valuable repository of data at the level of individual glyphs, which may be used for a variety of future investigations. One promising line of inquiry may be how script evolution interacts with the sound-symbolic properties of scripts, and whether different lineages encode different patterns of iconicity. Writing systems show clear chains of development over time, with more closely related scripts looking more similar to each other. To give examples from the scripts used in the study, the letters /i/ and /u/ share some overall similarities in the scripts from the Devanagari family: Devanagari and Bengali (IDs 17 and 36, respectively), as do scripts from the Arabic family, including Urdu, Sindhi, Book Pahlavi, Nestorian Syriac, Avestan and Parthian inscription (IDs 52, 24, 26, 35, 39, 37), albeit at different degrees of visual similarity. The norms for script guessability allow more fine-grained investigation of these relationships over historical time and geographical space.

## 7. Material and methods

### 7.1. Stimuli: ancient and unfamiliar letters

Stimuli were derived through a systematic review: letters representing /i/ and /u/ were collated from every script in the academic compendium *The World's Writing Systems* [81], the first publication of its kind to devote a chapter to each script, written by a leading expert in the field, which contains not only letter forms and letter names, but also detailed descriptions of the phonological systems of the languages and how each letter canonically mapped to different phonemes. In the stimulus set, we included all writing systems in which /i/ and /u/ could be written alone, represented by a single glyph. Scripts were excluded if they did not allow direct phonological representation without recourse to meaning (i.e. Chinese), or if they did not allow vowels to be represented (i.e. standard Hebrew). Since the task would involve decisions about individual vowels in isolation, for writing systems that employ position-dependent vowel representations, word-initial glyphs were selected. To maximize the acoustic contrast between /i/ and /u/, if scripts encoded tense/lax distinctions between high vowels, the tense form (the most peripheral) was selected. If the writing system encoded vowel length using reduplication or vowel plus a modifier, the unduplicated base form was selected (the short form). To avoid associations based on familiarity with the Latin script via English or other European languages, scripts from the Phoenician family (including Greek, Latin, Runic and Cyrillic) were excluded from the test set due to the familiarity of their ‘stick-and-bucket’ /i/ and /u/ letter forms. Finally, scripts were excluded if they duplicated a glyph-pair from another script in the test set (i.e. several scripts from the Arabic family). This procedure resulted in 56 distinct letter pairs from different scripts. The letters were photographed from the printed book and enlarged to form the visual stimuli of the following studies. The full set of glyphs is available in the Open Science Framework repository for this project (https://osf.io/xufmd).

### 7.2. Experiment 1: participants and procedure

Participants were 98 university students at the second author's institution in Singapore (ages 18–20: 29, ages 21–30: 68, 31–40: 1; female: 61.2%), recruited on campus, in an informal setting. All participants indicated they were over 18 and agreed to participate in the experiment. Two additional participants were tested but removed from analysis, as they did not reside in Singapore. Typical of the Singapore language environment, most participants were bilingual speakers of English and Mandarin Chinese (83.7%), along with bilingual speakers of English and Malay (8.3%), English and Tamil (2%), English and Korean (2%), English and French (2%) and monolingual English speakers (2%). It should be noted that bilingual speakers of Chinese have experience with an ideographic writing system, while speakers of Korean and Tamil have experience with syllabic writing systems, and also speakers of Malay and French use only the Latin alphabet. All were undertaking their university studies in English and spoke English at high levels of proficiency. Participants were given a printed page containing the 56 pairs of letters, arranged in four columns, in a pseudo-randomized order (figure 1*a*). Each column contained 14 letter pairs, with /i/ and /u/ counterbalanced for side of presentation. Participants were told that each pair contained the letter for the ‘ee’ sound in ‘feet’ and the ‘oo’ sound in ‘shoe’, and were asked to circle the ‘oo’ letter in each pair, in a paired 2-alternative forced choice (paired-2AFC) task. Singaporean English tends to have short/long vowel contrasts (/i/ versus /iː/, /u/ versus /uː/), rather than tense/lax (/i/ versus /I/, /u/ versus / ℧/) [82], meaning that the vowels in the example words are highly contrastive, as in other varieties of English. Participants were also instructed to mark any writing system they were familiar with, and to name it if they could. The entire procedure lasted less than 15 min. This research was approved by the Institutional Review Board of the second author's institution. Statistics were conducted using SPSS with Power analysis in G*Power [83].

### 7.3. Experiment 2: participants and procedure

Power analysis of the one-sample two-tailed *t*-test in Experiment 1 revealed that further replications would require a minimum sample of 59 participants for a high-powered replication [83]. Since our online sample was likely to be more diverse than our pencil-and-paper test, we aimed to recruit 80–100 participants per testing wave. In each wave, we stopped recruitment on a fixed date after 80 participants successfully completed the task. The photographed images used in the stimulus set for Experiment 1 were converted to 60 × 60 pixel png files for display in Qualtrics. In the first wave of the online study, participants guessed which letter in a pair was /i/, and in the second wave, a different group of participants guessed /u/. We also collected information on language use, asking participants to list the languages they spoke, and to rate how well they spoke them, on a five-point scale. Participants were 183 Internet users who took part in the test in English (/i/: *N* = 102; /u/: *N* *=* 81), were over the age of 18 (18–20: 13.7%, 21–30: 44.8%, 31–40: 25.1%, 41–50: 8.2%, 51–60: 6.6%, over 60: 1.1%, prefer not to say: 0.5%) and agreed to participate in the experiment (female: 56.3%, male: 39.9%; prefer not to say: 3.8%). All participants were speakers of English, and in addition, reported that they were from a variety of countries (Singapore (22.4%), Germany (21.3%), USA (19.7%), Serbia (12.6%), UK (4.4%), Austria and Australia (both 3.8%), The Netherlands (2.2%), Switzerland, Canada and Hungary (all 1.6%), France (1.1%), Malaysia, Italy, Japan, Finland, New Zealand, Mexico and Ireland (all 0.5%)) and spoke a variety of languages. Languages that received the highest self-report rating included English (52.7%), German (19.4%), Serbian (8.1%), Mandarin Chinese (3.7%), Croatian, Spanish and French (all 1.8%), Hungarian, Bosnian and Dutch (all 1.1%), Malay, Austrian German and Italian (all 0.7%), Cantonese, Russian, Hindi, Hebrew, Greek, Portuguese, Singlish (Singaporean English Creole), Czech, Finnish, Arabic, Bulgarian, Sinhala, Esperanto and Montenegrin (all 0.4%), as well as other languages (less than 0.04% each). It should be noted that some of the participants spoke languages which use only the Latin alphabet, while others reported speaking languages with a more diverse range of scripts. Full data are available in the Open Science Repository for this project (https://osf.io/xufmd). An additional 122 people began the study, but did not complete it (/i/ condition: 46, /u/ condition: 76). Incomplete responses were removed before analysis.

This research was approved by the Institutional Review Board. Participants were directed to the survey via the authors' lab website. After selecting ‘I am over 18 and I agree to take part in the study’, the procedure was the same as in Experiment 1, with the exception that each page of the online study included four letter pairs per page, pages were presented in a random order, and partial feedback and encouragement were given after every page of completed responses. To encourage people to complete both the quiz and demographic questions, final scores were displayed after demographic questions had been completed.

### 7.4. Experiment 3: procedure

Each image was a black glyph on a white background of 60 × 60 pixels. The png files derived from the original photographs of each letter were converted to grayscale for processing. To obtain the spatial frequency distribution for each image, we used a Fourier transform to convert the two-dimensional images into one-dimensional projections, using a custom script in Matlab 8.6 [84]. Each projection comprised the frequency of occurrence at which particular spatial frequencies were measured within a given image. The script is available in the OSF repository [38]. Spatial frequency distributions were computed for each letter separately.

## Data accessibility

Glyph files used as stimuli are available in the Open Science Framework repository for this project, along with the norms for glyph guessing, the spatial frequency code and the raw data for both glyph guessing and spatial frequency computations (https://osf.io/xufmd).

## References

[1] Perniss P, Vigliocco G 2014 The bridge of iconicity: from a world of experience to the experience of language. Phil. Trans. R. Soc. B 369, 20130300 (doi:10.1098/rstb.2013.0300) 25092668 10.1098/rstb.2013.0300 PMC4123679

[2] Dingemanse M, Blasi DE, Lupyan G, Christiansen MH, Monaghan P 2015 Arbitrariness, iconicity, and systematicity in language. Trends Cogn. Sci. 19, 603–615. (doi:10.1016/j.tics.2015.07.013) 26412098 10.1016/j.tics.2015.07.013

[3] de Saussure F 1911:1959 Course in general linguistics. New York, NY: Philosophical Library.

[4] Sapir E 1929 A study in phonetic symbolism. J. Exp. Psychol. 12, 225–239. (doi:10.1037/h0070931)

[5] Köhler W 1929 Gestalt psychology. New York, NY: Liveright Publishing Corporation.

[6] Holland MK, Wertheimer M 1964 Some physiognomic aspects of naming, or, maluma and takete revisited. Percept. Mot. Skills 19, 111–117. (doi:10.2466/pms.1964.19.1.111) 14197433 10.2466/pms.1964.19.1.111

[7] Ramachandran VS, Hubbard EM 2001 Synaesthesia—a window into perception, thought and language. J. Conscious. Stud. 8, 123–134.

[8] Tarte RD, Barritt LS 1971 Phonetic symbolism in adult native speakers of English: three studies. Lang. Speech 14, 158–168. (doi:10.1177/002383097101400206) 5566128 10.1177/002383097101400206

[9] Monaghan P, Shillcock RC, Christiansen MH, Kirby S 2014 How arbitrary is language? Phil. Trans. R. Soc. B 369, 20130299 (doi:10.1098/rstb.2013.0299) 25092667 10.1098/rstb.2013.0299 PMC4123678

[10] Kovic V, Plunkett K, Westermann G 2010 The shape of words in the brain. Cognition 114, 19–28. (doi:10.1016/j.cognition.2009.08.016) 19828141 10.1016/j.cognition.2009.08.016

[11] Parise CV, Spence C 2012 Audiovisual crossmodal correspondences and sound symbolism: a study using the implicit association test. Exp. Brain Res. 220, 319–333. (doi:10.1007/s00221-012-3140-6) 22706551 10.1007/s00221-012-3140-6

[12] Sučević J, Savić AM, Popović MB, Styles SJ, Ković V 2015 Balloons and bavoons vs spikes and shikes: ERPs reveal shared neural processes for shape-sound-meaning congruence in words, and shape-sound congruence in pseudowords. Brain Lang. 145–146, 11–22. (doi:10.1016/j.bandl.2015.03.011) 10.1016/j.bandl.2015.03.011 25935826

[13] Hung S-M, Styles SJ, Hsieh P-J 2017 Can a word sound like a shape before you have seen it? Sound-shape mapping prior to conscious awareness. Psychol. Sci. 28, 263–275. (doi:10.1177/0956797616677313) 28112997 10.1177/0956797616677313

[14] Westbury C 2005 Implicit sound symbolism in lexical access: evidence from an interference task. Brain Lang. 93, 10–19. (doi:10.1016/j.bandl.2004.07.006) 15766764 10.1016/j.bandl.2004.07.006

[15] Sidhu DM, Pexman PM 2016 A prime example of the Maluma/Takete effect? Testing for sound symbolic priming. Cogn. Sci. Early View, 1–30. 10.1111/cogs.12438 27766662

[16] Bremner AJ et al. 2013 ‘Bouba’ and ‘Kiki’ in Namibia? A remote culture make similar shape–sound matches, but different shape–taste matches to Westerners. Cognition 126, 165–172. (doi:10.1016/j.cognition.2012.09.007) 23121711 10.1016/j.cognition.2012.09.007

[17] Imai M, Kita S, Nagumo M, Okada H 2008 Sound symbolism between a word and an action facilitates early verb learning. Cognition 109, 54–65. (doi:10.1016/j.cognition.2008.07.015) 18835600 10.1016/j.cognition.2008.07.015

[18] Kantartzis K, Imai M, Kita S 2011 Japanese sound-symbolism facilitates word learning in English-speaking children. Cogn. Sci. 35, 575–586. (doi:10.1111/j.1551-6709.2010.01169.x)

[19] Maurer D, Pathman T, Mondloch CJ 2006 The shape of boubas: sound–shape correspondences in toddlers and adults. Dev. Sci. 9, 316–322. (doi:10.1111/j.1467-7687.2006.00495.x) 16669803 10.1111/j.1467-7687.2006.00495.x

[20] Spector F, Maurer D 2013 Early sound symbolism for vowel sounds. i-Perception 4, 239–241. (doi:10.1068/i0535) 24349684 10.1068/i0535 PMC3859567

[21] Ozturk O, Krehm M, Vouloumanos A 2013 Sound symbolism in infancy: evidence for sound–shape cross-modal correspondences in 4-month-olds. J. Exp. Child Psychol. 114, 173–186. (doi:10.1016/j.jecp.2012.05.004) 22960203 10.1016/j.jecp.2012.05.004

[22] Peña M, Mehler J, Nespor M 2011 The role of audiovisual processing in early conceptual development. Psychol. Sci. 22, 1419 (doi:10.1177/0956797611421791) 21960249 10.1177/0956797611421791

[23] Maurer D 1993 Neonatal synesthesia: implications for the processing of speech and faces. In Developmental neurocognition: speech and face processing in the first year of life (eds de Boysson-Bardies B, Jusczyk P, MacNeilage P, Morton J, de Schonen S), pp. 109–124. Dordrecht, The Netherlands: Kluver.

[24] Marks LE, Hammeal RJ, Bornstein MH 1987 Perceiving similarity and comprehending metaphor. Monogr. Soc. Res. Child 215, 1–102. (doi:10.2307/1166084) 3431563

[25] Cuskley C, Simner J, Kirby S 2015 Phonological and orthographic influences in the bouba–kiki effect. Psychol. Res. 24, 1–12. 10.1007/s00426-015-0709-2 26403463

[26] Doyle JR, Bottomley PA 2011 Mixed messages in brand names: separating the impacts of letter shape from sound symbolism. Psychol. Mark. 28, 749–762. (doi:10.1002/mar.20410)

[27] Kelly BF, Leben WR, Cohen RH 2003 The meanings of consonants. In Twenty-ninth Annual Meeting of the Berkeley Linguistics Society: General Session and Parasession on Phonetic Sources of Phonological Pattern: Synchronic and Diachronic Explanations, Berkeley, CA, 14–17 February, pp. 245–253. Berkeley Linguistics Society.

[28] Rogers SK, Ross AS 1975 A cross-cultural test of the Maluma-Takete phenomenon. Perception 4, 105–106. (doi:10.1068/p040105) 1161435 10.1068/p040105

[29] Koriat A, Levy I 1977 The symbolic implications of vowels and their orthographic representations in two natural languages. J. Psycholinguist. Res. 6, 93–104. (doi:10.1007/BF01074374)

[30] Nielsen AKS 2011 Sound symbolism and the Bouba-Kiki effect: uniting function and mechanism in the search for language universals. MSc thesis, University of Lethbridge, Lethbridge, Alberta, Canada.

[31] Ladefoged P 1993 A course in phonetics, 3rd edn Orlando, FL: Harcourt Brace.

[32] Moran S, McCloy D, Wright R 2014 PHOIBLE online. Leipzig, Germany: Max Planck Institute for Evolutionary Anthropology.

[33] Fort M, Martin A, Peperkamp S 2015 Consonants are more important than vowels in the Bouba-Kiki effect. Lang. Speech 58, 247–266. (doi:10.1177/0023830914534951) 26677645 10.1177/0023830914534951

[34] Styles SJ 2014 Psychoacoustic correlates of speech-sound/shape/size mapping in a 121-alternative forced-choice task. Figshare (International Multisensory Research Forum). https://dx.doi.org/10.6084/m9.figshare.3467909.

[35] D'Onofrio A 2014 Phonetic detail and dimensionality in sound-shape correspondences: refining the Bouba-Kiki paradigm. Lang. Speech 57, 367–393. (doi:10.1177/0023830913507694)

[36] Nielsen AKS, Rendall D 2013 Parsing the role of consonants versus vowels in the classic Takete-Maluma phenomenon. Can. J. Exp. Psychol. 67, 153–163. (doi:10.1037/a0030553) 23205509 10.1037/a0030553

[37] Ohala JJ 1994 The frequency code underlies the sound-symbolic use of voice pitch. In Sound symbolism (eds Hinton L, Nichols J, Ohala JJ), pp. 325–347. Cambridge, UK: Cambridge University Press.

[38] Turoman N, Styles SJ 2017 Ancient, unfamiliar letter-pairs for /i/ and /u/ with norms for letter guessing. (doi:10.17605/OSF.IO/XUFMD) (Open Science Framework). 10.1098/rsos.170882 PMC5627124 28989784

[39] Styles SJ 2014 What can ancient and unfamiliar scripts tell us about sound symbolism? Poster presented at the *Int. Multisensory Research Forum, Amsterdam, The Netherlands, June*.

[40] Evans KK, Treisman A 2010 Natural cross-modal mappings between visual and auditory features. J. Vis. 10, 1–12. (doi:10.1167/10.7.1263) 10.1167/10.1.6 PMC2920420 20143899

[41] Mondloch C, Maurer D 2004 Do small white balls squeak? Pitch-object correspondences in young children. Cogn. Affect. Behav. Neurosci. 4, 133–136. (doi:10.3758/CABN.4.2.133) 15460920 10.3758/cabn.4.2.133

[42] Spence C, Gallace A 2011 Tasting shapes and words. Food Qual. Preference 22, 290–295. (doi:10.1016/j.foodqual.2010.11.005)

[43] Marks LE 1987 On cross-modal similarity: auditory–visual interactions in speeded discrimination. J. Exp. Psychol. Human Percept. Perform. 13, 384 (doi:10.1037/0096-1523.13.3.384) 2958587 10.1037//0096-1523.13.3.384

[44] Lewkowicz DJ, Turkewitz G 1980 Cross-modal equivalence in early infancy: auditory–visual intensity matching. Dev. Psychol. 16, 597 (doi:10.1037/0012-1649.16.6.597)

[45] Walker P et al. 2010 Preverbal infants' sensitivity to synaesthetic cross-modality correspondences. Psychol. Sci. 21, 21–25. (doi:10.1177/0956797609354734) 20424017 10.1177/0956797609354734

[46] Dolscheid S, Hunnius S, Casasanto D, Majid A 2014 Prelinguistic infants are sensitive to space-pitch associations found across cultures. Psychol. Sci. 25, 1256–1261. (doi:10.1177/0956797614528521) 24899170 10.1177/0956797614528521

[47] Maurer D 1993 Neonatal synesthesia: implications for the processing of speech and faces. In Developmental neurocognition: speech and face processing in the first year of life (eds B de Boysson-Bardies, S de Schonen, P Jusczyk, P McNeilage, J Morton), pp. 109–124. Dordrecht, The Netherlands: Springer (doi:10.1007/978-94-015-8234-6_10)

[48] Ludwig VU, Adachi I, Matsuzawa T 2011 Visuoauditory mappings between high luminance and high pitch are shared by chimpanzees (*Pan troglodytes*) and humans. Proc. Natl Acad. Sci. USA 108, 20 661–20 665. (doi:10.1073/pnas.1112605108) 10.1073/pnas.1112605108 PMC3251154 22143791

[49] Revill KP, Namy LL, DeFife LC, Nygaard LC 2014 Cross-linguistic sound symbolism and crossmodal correspondence: evidence from fMRI and DTI. Brain Lang. 128, 18–24. (doi:10.1016/j.bandl.2013.11.002) 24316238 10.1016/j.bandl.2013.11.002

[50] Stein BE, Stanford TR, Rowland BA 2014 Development of multisensory integration from the perspective of the individual neuron. Nat. Rev. Neurosci. 15, 520–535. (doi:10.1038/nrn3742) 25158358 10.1038/nrn3742 PMC4215474

[51] Morton EW 1977 On the occurrence and significance of motivation-structural rules in some bird and mammal sounds. Am. Nat. 111, 855–869. (doi:10.1086/283219)

[52] Bee M, Perrill S, Owen PC 2000 Male green frogs lower the pitch of acoustic signals in defense of territories: a possible dishonest signal of size? Behav. Ecol. 11, 169–177. (doi:10.1093/beheco/11.2.169)

[53] Coward S, Stevens CJ 2004 Extracting meaning from sound: nomic mappings, everyday listening, and perceiving object size from frequency. Psychol. Record 54, 349–364. (doi:10.1007/BF03395478)

[54] Gaver WW 1993 How do we hear in the world? Explorations in ecological acoustics. Ecol. Psychol. 5, 285–313. (doi:10.1207/s15326969eco0504_2)

[55] Spence C, Zampini M 2006 Auditory contributions to multisensory product perception. Acta Acust. United Acust. 92, 1009–1025.

[56] Styles SJ, Gawne L 2017 When does maluma/takete fail? Two key failures and a meta-analysis suggest that phonology and phonotactics matter. i-Perception 8, 1–17. (doi:10.1111/desc.12157) 10.1177/2041669517724807 PMC5574486 28890777

[57] Dolscheid S, Shayan S, Majid A, Casasanto D 2013 The thickness of musical pitch: psychophysical evidence for linguistic relativity. Psychol. Sci. 24, 613–621. (doi:10.1177/0956797612457374) 23538914 10.1177/0956797612457374

[58] Shayan S, Ozturk O, Bowerman M, Majid A 2014 Spatial metaphor in language can promote the development of cross-modal mappings in children. Dev. Psychol. 17, 636–643. 10.1111/desc.12157 24636133

[59] Werker JF, Tees RC 1984 Cross-language speech perception: evidence for perceptual reorganization during the first year of life. Infant Behav. Develop. 7, 49–63. (doi:10.1016/S0163-6383(84)80022-3)

[60] Kuhl PK 2004 Early language acquisition: cracking the speech code. Nat. Rev. Neurosci. 5, 831–843. (doi:10.1038/nrn1533) 15496861 10.1038/nrn1533

[61] Polka L, Bohn O-S 2003 Asymmetries in vowel perception. Speech Commun. 41, 221–231. (doi:10.1016/S0167-6393(02)00105-X)

[62] Taylor IK 1963 Phonetic symbolism re-examined. Psychol. Bull. 60, 200–209. (doi:10.1037/h0040632) 13993599 10.1037/h0040632

[63] Taylor IK, Taylor MM 1965 Another look at phonetic symbolism. Psychol. Bull. 64, 413–427. (doi:10.1037/h0022737) 4159231 10.1037/h0022737

[64] Henrich J, Heine SJ, Norenzayan A 2010 The weirdest people in the world? Behav. Brain Sci. 33, 61–135. (doi:10.1017/S0140525X0999152X) 20550733 10.1017/S0140525X0999152X

[65] Nielsen AKS, Rendall D 2012 The source and magnitude of sound-symbolic biases in processing artificial word material and their implications for language learning and transmission. Lang. Cogn. 4, 115–125. (doi:10.1515/langcog-2012-0007)

[66] Chen Y, Huang P, Woods A, Spence C 2016 When ‘Bouba’ equals ‘Kiki’: cultural commonalities and cultural differences in sound-shape correspondences. Sci. Rep. 27, 26681 (doi:10.1038/srep26681) 10.1038/srep26681 PMC4882484 27230754

[67] Dehaene S, Cohen L, Sigman M, Vinckier F 2005 The neural code for written words: a proposal. Trends Cogn. Sci. 9, 335–341. (doi:10.1016/j.tics.2005.05.004) 15951224 10.1016/j.tics.2005.05.004

[68] Sean D 2005 Some demographic and socio-cultural aspects of synesthesia. In Synesthesia: perspectives from cognitive neuroscience (eds Robertson L, Sagiv N), pp. 11–33. Oxford, UK: Oxford University Press.

[69] Simner J et al. 2006 Synaesthesia: the prevalence of atypical cross-modal experiences. Perception 35, 1024–1033. (doi:10.1068/p5469) 17076063 10.1068/p5469

[70] Bankieris K, Simner J 2015 What is the link between synaesthesia and sound symbolism? Cognition 136, 186–195. (doi:10.1016/j.cognition.2014.11.013) 25498744 10.1016/j.cognition.2014.11.013 PMC4415500

[71] Cytowic RE, Eagleman DM 2009 Wednesday is indigo blue: discovering the brain of synesthesia. Cambridge, UK: MIT Press.

[72] Karipidis II et al. 2017 Neural initialization of audiovisual integration in prereaders at varying risk for developmental dyslexia. Hum. Brain Mapp. 38, 1038–1055. (doi:10.1002/hbm.23437) 27739608 10.1002/hbm.23437 PMC6866885

[73] Styles SJ 2016 The language of dance: testing a model of cross-modal communication in the performing arts. In ICMA array (eds Lindborg PM, Styles SJ), pp. 35–42. Special Issue: Proceedings of Si15, Singapore August 2015.

[74] Vandermosten M et al. 2012 A tractography study in dyslexia: neuroanatomic correlates of orthographic, phonological and speech processing. Brain 135, 935–948. (doi:10.1093/brain/awr363) 22327793 10.1093/brain/awr363

[75] Drijvers L, Zaadnoordijk L, Dingemanse M 2015 Sound-symbolism is disrupted in dyslexia: implications for the role of cross-modal abstraction processes. In 37th Annual Meeting of the Cognitive Science Society (eds Noelle D, Dale R, Warlaumont AS, Yoshimi J, Matlock T, Jennings CD). Austin, TX: Cognitive Science Society.

[76] Snowling MJ 1980 The development of grapheme-phoneme correspondence in normal and dyslexic readers. J. Exp. Child Psychol. 29, 294–305. (doi:10.1016/0022-0965(80)90021-1) 7365427 10.1016/0022-0965(80)90021-1

[77] Blomert L 2011 The neural signature of orthographic-phonological binding in successful and failing reading development. Neuroimage 57, 695–703. (doi:10.1016/j.neuroimage.2010.11.003) 21056673 10.1016/j.neuroimage.2010.11.003

[78] Brem S et al. 2010 Brain sensitivity to print emerges when children learn letter-speech sound correspondences. Proc. Natl Acad. Sci. USA 107, 7939–7944. (doi:10.1073/pnas.0904402107) 20395549 10.1073/pnas.0904402107 PMC2867899

[79] Perniss P, Thompson RL, Vigliocco G 2010 Iconicity as a general property of language: evidence from spoken and signed languages. Front. Psychol. 1, 1–15. (doi:10.3389/fpsyg.2010.00227) 21833282 10.3389/fpsyg.2010.00227 PMC3153832

[80] Ković V, Sučević J, Styles SJ 2017 To call a cloud 'cirrus': sound symbolism in names for categories or items. PeerJ 5, 1–18. (doi:10.7717/peerj.3466) 10.7717/peerj.3466 PMC5493972 28674648

[81] Daniels PT, Bright W 1996 The world's writing systems. Oxford, UK: Oxford University Press.

[82] Deterding D 2003 An instrumental study of the monophthong vowels of Singapore English. English World-Wide 24, 1–6. (doi:10.1075/eww.24.1.02det)

[83] Faul F, Erdfelder E, Lang A-G, Buchner A 2007 G*Power 3: a flexible statistical power analysis program for the social, behavioral, and biomedical sciences. Behav. Res. Methods 39, 175–191. (doi:10.3758/BF03193146) 17695343 10.3758/bf03193146

[84] Matlab. 2015 Natick, MA: The MathWorks Inc., 8.6.

[85] Turoman N, Styles SJ 2016 How well do humans capture the sounds of speech in writing? In ICMA array (eds Lindborg PM, Styles SJ), pp. 43–44. Special Issue: Proceedings of Si15, Singapore, August 2015.

